# Social Determinants and Barriers to Health Insurance Utilization Among the Rural Population: A Mixed-Methods Study in a Rural Field Practice Area of Tiruvallur District, Tamil Nadu

**DOI:** 10.7759/cureus.108245

**Published:** 2026-05-04

**Authors:** M Vinoth Kumar, Seshadhri A, Gudluru Uday Sai

**Affiliations:** 1 Community Medicine, Sri Venkateswaraa Medical College Hospital and Research Institute, Sri Venkateswaraa University, Chennai, IND

**Keywords:** healthcare barriers, healthcare utilization, health insurance, out-of-pocket expenditure, rural population

## Abstract

Background: Health insurance plays a crucial role in reducing out-of-pocket healthcare expenditure, improving access to healthcare services particularly in rural populations. The disparities in health insurance schemes still exist in India despite the presence of publicly funded health insurance schemes, more especially among the socially disadvantaged groups. This study aimed to assess the level of health insurance utilization and its association with selected social determinants and to explore perceived barriers and facilitators influencing utilization using a mixed-methods approach.

Methods: The study was done in the rural field practice area of Tiruvallur district, Tamil Nadu, through a community-based mixed-methods approach. The quantitative arm comprised 200 adults aged ≥18 years with a history of hospitalization within the past year using a systematic random sampling technique. The information for the study was collected through household interviews by using a structured questionnaire and analyzed using IBM SPSS Statistics for Windows, Version 25.0 (IBM Corp., Armonk, New York, United States), with the help of descriptive statistics and chi-squared tests. The qualitative component consisted of 30 purposively selected participants and caregivers who took part in in-depth interviews until thematic saturation was reached. Thematic analysis was done to come up with the key barriers to health insurance utilization.

Results: Out of 200 respondents, only 56 people (28%) reported using health insurance services, and 144 people (72%) did not use available insurance. The government-sponsored schemes accounted for the highest utilization (75%). Awareness of insurance schemes was reported by 112 participants (56%). Significant associations were observed between utilization of insurance and education level (p=0.001), socioeconomic status (p=0.002), awareness (p<0.001), occupation (p=0.01), type of illness (p=0.003), and distance to the healthcare facilities (p=0.02). Limited awareness, administrative complexities, indirect costs, sociocultural influences, and health system constraints were the major barriers as per qualitative findings.

Conclusion: Health insurance utilization in rural settings remains limited due to several social and systemic factors. Community awareness, the simplification of administrative procedures, and improved healthcare accessibility are essential to help realize its effective utilization and thus support efforts toward universal health coverage.

## Introduction

Access to equitable and affordable healthcare remains a major public health challenge globally, particularly in low- and middle-income countries (LMICs), where out-of-pocket expenditure forms a significant proportion of health spending [1,2]. Health insurance has been identified as an important approach to cushion against financial distress and to enhance equitable access to healthcare services particularly for vulnerable groups [2,3]. Different publicly funded health insurance schemes have been introduced in India to promote the goal of universal health coverage, but disparities in utilization persist [4,5].

Rural populations in India have unique challenges in accessing healthcare services, limited health infrastructure, low socioeconomic status, and limited awareness of available health schemes [6,7]. Caste, education, occupation, income, and place of residence are social determinants of health that greatly affect health-seeking attitude and healthcare usage [7,8]. Rural residents are generally less likely to utilize health insurance services despite being equally eligible under various government health insurance schemes as their urban counterparts [6,9]. Sociocultural factors are also critical in influencing healthcare utilization in rural communities. Gender norms, family decision-making dynamics, and cultural beliefs often play a role in determining access to healthcare services with specific regards to women and elderly persons. Other than that, perceptions on quality of care under insurance schemes and trust in public healthcare systems may further influence utilization [10,11].

Healthcare utilization in rural populations is influenced by social, economic, and healthcare access factors. Andersen's behavioral model describes healthcare utilization as being shaped by predisposing, enabling, and need-based determinants, including factors such as education, socioeconomic status, awareness, and accessibility of services [12]. In the present rural field practice population of Tiruvallur district, similar determinants such as education level, socioeconomic status, awareness of health insurance schemes, distance to healthcare facilities, and severity of illness were found to influence utilization patterns. These findings highlight that healthcare utilization in rural communities is determined by a combination of social characteristics and access-related factors that affect the ability to use available insurance services [8,13,14].

In India, several studies have reported that health insurance coverage alone does not necessarily translate into actual utilization, especially in underserved populations [5,15]. Common barriers include a lack of adequate knowledge about insurance schemes, administrative complexities, documentation requirements, and indirect costs related to healthcare, transportation, and wage loss [14,16]. The barriers are tilted against the socially marginalized groups, further exacerbating health inequities [7,16].

In spite of the growing number of literature on health insurance in India, there are a limited number of studies with references on rural field practice areas where community-level factors and lived experiences are critical in influencing care utilization patterns [6,13]. For one, most of the reviewed literature leaned on quantitative data, thus limiting context-specific barriers and facilitators. A mixed-methods approach is necessary to quantitatively identify the determinants and to qualitatively explore the contextual barriers and facilitators of health insurance utilization in rural India [1].

The present study aimed to assess the level of health insurance utilization and its association with selected social determinants among adults in a rural field practice area of Tiruvallur district, Tamil Nadu, and to explore perceived barriers and facilitators influencing health insurance utilization through qualitative interviews using a mixed-methods approach.

## Materials and methods

Study design and setting

A mixed-methods study was conducted to assess the social determinants and barriers to health insurance utilization. The study was carried out in the rural field practice area, Ponneri, attached to Sri Venkateswaraa Medical College Hospital and Research Institute, located in Tiruvallur district, Tamil Nadu, India. 

Study period

The study was conducted over a period of four months from December 2025 to March 2026.

Study population

The quantitative component included adult residents from the rural field practice area who had been hospitalized within the past one year. The qualitative component included a purposive sample of participants and their primary caregivers who had experienced either successful or unsuccessful utilization of health insurance during hospitalization. This facilitated an in-depth exploration of perceived facilitators and barriers from the community perspective. The population register used for sampling was prepared using hospitalization records obtained from the rural field practice area health center. Eligible individuals were identified from records of hospital admissions during the previous one year. The list was verified and updated through household visits to ensure accuracy before applying the sampling procedure.

Inclusion criteria

For the quantitative component, individuals aged 18 years and above residing in the rural field practice area who had a history of inpatient admission within the past one year and were able to provide relevant information on health insurance status and social determinants (age, gender, education, occupation, income, caste, and place of residence) were included.

For the qualitative component, participants or their primary caregivers aged 18 years and above who had experienced hospitalization and were willing to provide informed consent were included. Participants who had either utilized or not utilized health insurance services during hospitalization were eligible. Additionally, participants were required to be able to communicate effectively in Tamil or English.

Exclusion criteria

Individuals who were unable to provide complete information regarding health insurance utilization or key social determinants were excluded from the quantitative component. Participants who were critically ill, cognitively impaired, or unable to participate in the interview process were excluded from the qualitative component. Individuals unwilling or unable to provide informed consent as well as caregivers not involved in healthcare decision-making or hospitalization processes were also excluded.

Sample size and sampling technique

The sample size calculation for the quantitative component involved the formula for determining the proportion with finite population correction. The total number of eligible adults with experience of being hospitalized within the last one year at the rural field practice area was N=400. With the assumption that the expected proportion (p) was 50%, confidence level at 95% (Z=1.96), and the margin of error (e) was 5%, the formula used to calculate the sample size was as follows: $\mathrm{n}=\left[\mathrm{Z}^{2}\times\mathrm{p}\times(1-\mathrm{p})/\mathrm{e}^{2}\right]/\left[1+(\mathrm{Z}^{2}\times\mathrm{p}\times(1-\mathrm{p})/(\mathrm{e}^{2}\times\mathrm{N}))\right]$.

Substituting the respective values in the formula (Z=1.96; p=0.5; e=0.05; N=400), the sample size obtained was 196. The sample size was rounded off to 200. The participants who were eligible were systematically sampled from the population registry of the rural field practice area. There were no exclusions among the eligible participants, and any necessary replacements were done to attain the sample size of 200 participants.

A household-based sampling approach was employed. Eligible participants were selected using systematic random sampling from the rural field practice area population register. In households with more than one eligible participant, one individual was selected using simple random sampling.

For the qualitative component, participants were selected using purposive sampling. Participants for the qualitative component included individuals representing diverse age groups, gender categories, and socioeconomic backgrounds to capture varied perspectives on health insurance utilization. In-depth interviews were conducted sequentially, and thematic saturation was assessed concurrently during data collection. After every five interviews, transcripts were reviewed to identify the emergence of new codes or themes. Thematic saturation was considered achieved when no new themes or subthemes emerged in three consecutive interviews. Saturation was observed after the 28th interview, and two additional interviews were conducted to confirm the stability of identified themes, resulting in a total of 30 interviews.

Development and validation of the questionnaire

The quantitative questionnaire was designed following a thorough review of existing literature pertaining to the utilization of health insurance and social determinants of health. Domains relevant to the topic of study, including socio-demographics, knowledge about health insurance programs, utilization, barriers to utilization, and cost incurred in seeking care, were selected and integrated into the questionnaire.

The preliminary version of the questionnaire was subjected to evaluation by three subject experts from Community Medicine to determine content validity and the appropriateness of the items. Modifications were made accordingly.

The questionnaire was piloted among 20 respondents living in a neighboring rural community that did not form part of the actual study sample to determine comprehension, reliability, and feasibility. Changes were made in terms of wordings and question ordering.

Reliability testing involved computation of the Cronbach's alpha coefficient, and any value of ≥0.7 was deemed satisfactory. The final questionnaire was used on the actual study respondents through the investigators. The internal consistency of the questionnaire was assessed using Cronbach's alpha, which was found to be 0.81, indicating acceptable reliability.

Data collection

Quantitative data were collected through household visits using a validated and pre-tested semi-structured questionnaire administered by trained investigators (Appendix A). The questionnaire captured information on socio-demographic characteristics, health insurance status, awareness, and utilization patterns. The questionnaire was interviewer-administered in the local language (Tamil). The original questionnaire was prepared in English and translated into Tamil followed by back-translation into English to ensure conceptual equivalence and linguistic accuracy.

For the qualitative component, in-depth interviews were conducted using a semi-structured interview guide developed based on a review of relevant literature (Appendix B). The guide was pilot-tested and refined prior to data collection. Interviews were conducted in Tamil or English, audio-recorded with consent, and supplemented by field notes. Each in-depth interview lasted approximately 30-45 minutes. Interviews were conducted at participants' homes, which are within the community, to ensure confidentiality and comfort.

Statistical analysis

Quantitative data were entered into Microsoft Excel (Microsoft Corporation, Redmond, Washington, United States) and analyzed using IBM SPSS Statistics for Windows, Version 25.0 (IBM Corp., Armonk, New York, United States). Descriptive statistics such as frequency, percentage, mean, and standard deviation were used to summarize the data. The chi-squared test was applied to assess associations between social determinants and health insurance utilization. A p-value of ≤0.05 was considered statistically significant. For qualitative analysis, audio-recorded interviews were transcribed verbatim and translated into English where necessary. Thematic analysis was performed manually by two independent investigators. Codes were generated and grouped into themes using standard qualitative research techniques. Any discrepancies between investigators were resolved through discussion and consensus. The findings were reported in accordance with the Consolidated Criteria for Reporting Qualitative Research (COREQ) guidelines. Thematic analysis was performed using an inductive coding approach, allowing themes to emerge from the data rather than being predetermined.

Ethical considerations

Ethical approval was obtained from the Institutional Ethics Committee of Sri Venkateswaraa Medical College Hospital and Research Institute (IEC-SVMCH&RI) (approval number: 007/12/2025/IEC/SVMCHRI) prior to the commencement of the study. Written informed consent was obtained from all participants before data collection. Confidentiality and anonymity of participants were strictly maintained throughout the study.

## Results

As shown in Table 1, 200 participants from the rural field practice area were included in the study. The majority of participants were in the 40-60-year age group (44%), followed by those aged 20-39 years (34.5%) and above 60 years (21.5%). There was a majority of males (54%) in the study population compared to females (46%). On education, 36% had secondary education, while 22.5% were illiterate. A large proportion of participants were engaged as unskilled or semi-skilled workers (47.5%), and 52% belonged to a lower socioeconomic status. 

**Table 1:** Socio-demographic characteristics of the participants (n=200)

Variable	Frequency (n)	Percentage (%)
Age group (years)		
20-39	69	34.5
40-60	88	44
>60	43	21.5
Gender		
Male	108	54
Female	92	46
Education		
Illiterate	45	22.5
Primary	53	26.5
Secondary	72	36
Higher	30	15
Occupation		
Unemployed	42	21
Unskilled/semi-skilled	95	47.5
Skilled	40	20
Professional	23	11.5
Socioeconomic status		
Lower	104	52
Middle	68	34
Upper	28	14

As shown in Table 2, out of 200 participants, 56 (28%) reported that they made use of health insurance, while the majority of participants, 144 (72%), had not made use of insurance services. Of those who utilized insurance (n=56), participants enrolled in government schemes were the majority (42; 75%), followed by private insurance (10; 17.9%) and employer insurance (4; 7.1%).

**Table 2:** Pattern of health insurance utilization (n=200)

Variable	Frequency (n)	Percentage (%)
Utilized insurance	56	28
Not utilized	144	72
Type of insurance (n=56)		
Government schemes	42	75
Private insurance	10	17.9
Employer-based	4	7.1

As shown in Table 3, among the total study participants, 112 (56%) were aware of available health insurance schemes, whereas 88 (44%) reported a lack of awareness regarding such schemes.

**Table 3:** Awareness of health insurance schemes (n=200)

Awareness status	Frequency (n)	Percentage (%)
Aware	112	56
Not aware	88	44

As shown in Table 4, there is a statistically significant relationship between education and health insurance utilization (χ²=12.49; p=0.001), whereby there is an increase from 17.8% utilization among participants who are illiterate to 50% among those who have higher education levels. There is also a statistically significant relationship between socioeconomic status and utilization (χ²=9.73; p=0.002), since 37.5% of participants in the middle/upper classes utilized their insurance, while only 19.2% of those in the lower class utilized theirs. Awareness showed a statistically significant relationship with utilization (χ²=14.85; p<0.001), since 37.5% utilized their insurance compared to 15.9% of those not aware.

**Table 4:** Association between selected variables and insurance utilization Chi-squared test was applied. * indicates statistically significant at p≤0.05.

Variable	Utilized n (%)	Not utilized n (%)	χ² value	P-value
Education				
Illiterate	8 (17.8)	37 (82.2)	12.49	0.001*
Primary	10 (18.8)	43 (81.2)		
Secondary	23 (31.9)	49 (68.1)		
Higher	15 (50)	15 (50)		
Socioeconomic status				
Lower	20 (19.2)	84 (80.8)	9.73	0.002*
Middle/upper	36 (37.5)	60 (62.5)		
Awareness				
Aware	42 (37.5)	70 (62.5)	14.85	<0.001*
Not aware	14 (15.9)	74 (84.1)		
Gender				
Male	30 (27.8)	78 (72.2)	0.01	0.91
Female	26 (28.3)	66 (71.7)		

As shown in Table 5, lack of awareness (50; 34.7%) was the most frequently cited reason among participants who did not use insurance (n=144), followed by procedural challenges (34; 23.6%), absence of necessary documentation (24; 16.7%), preference for direct payment (20; 13.9%), and other reasons (16; 11.1%).

**Table 5:** Reasons for non-utilization (n=144)

Reason	Frequency (n)	Percentage (%)
Lack of awareness	50	34.7
Procedural difficulties	34	23.6
Lack of documents	24	16.7
Preference for direct payment	20	13.9
Other reasons	16	11.1

As shown in Table 6, family or friends were the most frequent source of information among participants (n=112) who were aware of health insurance schemes (40; 35.7%), followed by health professionals like accredited social health activist (ASHA)/auxiliary nurse midwife (ANM) (29; 25.9%), media sources like radio or television (25; 22.3%), and other sources (18; 16.1%).

**Table 6:** Source of information about health insurance (n=112) ASHA: accredited social health activist; ANM: auxiliary nurse midwife

Source	Frequency (n)	Percentage (%)
Family/friends	40	35.7
Health workers (ASHA/ANM)	29	25.9
Media (TV/radio)	25	22.3
Others	18	16.1

As shown in Table 7, the type of illness and insurance utilization were found to be statistically significantly correlated (p=0.003). Compared to participants with minor illnesses (18; 16.8%), those with major illnesses demonstrated higher utilization (38; 40.4%).

**Table 7:** Type of illness vs. insurance utilization Chi-squared test was applied. * indicates statistically significant at p≤0.05.

Type of illness	Utilized n (%)	Not utilized n (%)	χ² value	P-value
Major illness	38 (40.4)	56 (59.6)	8.78	0.003*
Minor illness	18 (16.8)	88 (83.2)		

As shown in Table 8, there was a statistically significant correlation between insurance utilization and the distance to a medical facility (p=0.02). Individuals who lived within 5 km showed higher utilization (32; 36.4%) than those who lived more than 5 km away (24; 21.4%).

**Table 8:** Distance to facility vs. insurance utilization Chi-squared test was applied. * indicates statistically significant at p≤0.05.

Distance	Utilized n (%)	Not utilized n (%)	χ² value	P-value
<5 km	32 (36.4)	56 (63.6)	5.34	0.02*
≥5 km	24 (21.4)	88 (78.6)		

As shown in Table 9, participants who were aware of insurance schemes were more likely to use government schemes (36; 85.7%) than private insurance (5; 11.9%) or employer-based insurance (1; 2.4%) among insurance users (n=56). Among those who were unaware, employer-based insurance (3; 21.4%) and private insurance (5; 35.7%) were used at comparatively higher rates than government programs (6; 42.9%).

**Table 9:** Awareness vs. type of insurance (n=56)

Awareness	Government n (%)	Private n (%)	Employer n (%)
Aware	36 (85.7)	5 (11.9)	1 (2.4)
Not aware	6 (42.9)	5 (35.7)	3 (21.4)

As shown in Table 10, the mean out-of-pocket expenditure among insured participants was ₹4,200 (SD=1,500), whereas uninsured participants incurred substantially higher mean expenditure of ₹9,800 (SD=2,300).

**Table 10:** Out-of-pocket expenditure comparison

Group	Mean expenditure (₹)	SD
Insured	4,200	1,500
Uninsured	9,800	2,300

As shown in Table 11, occupation demonstrated a statistically significant association with insurance utilization (p=0.01). Participants engaged in skilled or professional occupations showed higher utilization (36; 36.7%) compared to those in unskilled occupations (20; 21.1%).

**Table 11:** Occupation vs. insurance utilization Chi-squared test was applied. * indicates statistically significant at p≤0.05.

Occupation	Utilized n (%)	Not utilized n (%)	χ² value	P-value
Unskilled	20 (21.1)	75 (78.9)	6.63	0.01*
Skilled/professional	36 (36.7)	62 (63.3)		

Qualitative results

The researchers conducted 30 in-depth interviews with participants and their main caregivers who lived in the rural field practice area. The participants showed different social and demographic characteristics through their age, gender, educational level, job, and income level. The qualitative research method revealed a complete understanding of how rural community residents use health insurance because it showed their views, real-life situations, and main obstacles to obtaining insurance. The thematic analysis of interview transcripts produced five main themes which emerged through this analysis process: (1) limited awareness and understanding of health insurance, (2) administrative and procedural barriers, (3) financial constraints and indirect costs, (4) sociocultural influences and decision-making, and (5) health system-related challenges. The themes demonstrate that multiple factors contribute to the low health insurance usage which the quantitative research showed.

Theme 1: Limited Awareness and Understanding of Health Insurance

The interviews revealed that rural participants had insufficient knowledge about health insurance programs which resulted in their restricted understanding of these programs. The participants who had heard about the government programs did not possess complete knowledge about requirements, program advantages, and how to use the programs. The participants reported that they did not know about their insurance benefits which resulted in their non-usage of benefits during their hospital stays. 

"We were not aware that this treatment was eligible for coverage under the government health scheme so we ended up paying the entire cost from our own pocket. No one informed us about the availability of financial support, and we only came to know about the scheme later from others. By that time, we had already borrowed money and faced financial strain to manage the treatment expenses."

In several cases, participants associated insurance schemes only with specific conditions or populations, leading to misconceptions and underutilization.

"We thought that insurance schemes were meant only for very poor people or for special cases and not for families like ours. Because of this belief, we never tried to apply for the scheme or ask whether we were eligible. We assumed that we would not qualify so we continued to manage the treatment expenses on our own even though it was financially difficult for us."

Participants with lower educational status and those from economically disadvantaged backgrounds demonstrated particularly low awareness levels. People in rural areas received information through informal channels which included their neighbors and relatives as their primary source of information. 

"We heard about the scheme from other people in the village, but no one explained it to us properly. We only had partial information and did not understand how to apply or what benefits were included. Because of the lack of clear guidance, we felt confused and unsure about the process so we did not take any steps to enroll or use the scheme."

Theme 2: Administrative and Procedural Barriers

Participants frequently reported administrative difficulties and complex procedures as major barriers to utilizing health insurance. The process of obtaining insurance benefits proved to be confusing and time-consuming for people who lacked knowledge about official procedures. People considered documentation requirements which included identity proof, income certificates, and insurance cards as obstacles to their progress. Many participants indicated that they did not possess the required documents at the time of hospitalization.

"They asked for many documents during the application process, and we did not have all the required papers ready at that time. Some of the documents were difficult to obtain, and we were not clearly informed beforehand about what was needed. Because of this delay and confusion, we were unable to complete the process on time and could not make use of the insurance for the treatment."

Additionally, participants described challenges in navigating hospital procedures including multiple steps, lack of clear instructions, and delays in approval.

"We had to go to different counters and wait for a long time to complete the formalities. The process was very time-consuming, and there was no clear guidance about where to go or what steps to follow. It was difficult for us to understand the procedure, and we felt confused and tired moving from one place to another. Because of this complicated process, we found it hard to use the insurance services smoothly."

Theme 3: Financial Constraints and Indirect Costs

Although health insurance is intended to reduce financial burden, participants highlighted the persistence of indirect costs and partial financial protection. Even among those who utilized insurance, expenses related to medications, diagnostic tests, transportation, and food were often not covered.

"Even though we had insurance, we still had to spend money from our own pocket for medicines and certain tests that were not available in the hospital. We were asked to buy medicines from outside pharmacies and undergo tests at private laboratories which increased our expenses. This made us feel that the insurance did not fully cover all the treatment costs and we still faced financial burden despite being enrolled in the scheme."

For daily wage workers, the loss of income during hospitalization was an additional concern influencing decisions regarding healthcare utilization.

"If we stay in the hospital for treatment, we lose our daily wages, and that becomes a big problem for our family. Since we depend on daily earnings, even missing a single day of work affects our income and makes it difficult to manage household expenses. Sometimes, we hesitate to stay in the hospital or continue treatment because we are worried about losing wages and not being able to support our family financially."

Some participants expressed that the perceived effort required to utilize insurance did not justify the benefits particularly for minor illnesses.

"For small treatments, it feels easier to pay the amount directly rather than go through all the procedures required to use the insurance. The process of applying, submitting documents, and waiting for approval takes time and effort which does not seem worthwhile for minor expenses. Because of this, we prefer to pay from our own pocket for smaller treatments instead of using the insurance benefits."

Theme 4: Sociocultural Influences and Decision-Making

Sociocultural factors played a crucial role in shaping healthcare-seeking behavior and insurance utilization in the rural community. Decision-making was often influenced by family dynamics, gender roles, and social norms.

Female participants frequently reported limited autonomy in healthcare decisions relying on male family members for financial and treatment-related choices.

"My husband usually makes all the decisions regarding treatment and financial matters so I do not know much about insurance schemes or how they work. I depend on him to handle the documents and communicate with the hospital staff. Because I am not directly involved in these matters, I have limited knowledge about insurance benefits and procedures."

Elderly participants also depended on younger family members for navigating healthcare services which sometimes delayed or prevented insurance utilization.

In some cases, stigma and perceptions associated with government schemes influenced behavior. A few participants expressed reluctance to use insurance due to fear of being perceived as economically disadvantaged.

"People may think that we are poor if we use government schemes, and that makes us feel hesitant to apply for them."

Trust in healthcare providers and perceived quality of care also affected utilization. Some participants believed that patients using insurance might receive inferior services.

"If we use insurance, they may not treat us properly, so we prefer to pay."

Theme 5: Health System-Related Challenges

Health system factors emerged as significant contributors to low utilization of health insurance. Participants reported issues such as limited availability of empanelled hospitals, lack of clarity regarding covered services, and delays in approval processes.

Some participants indicated that not all treatments were covered under insurance schemes leading to unexpected out-of-pocket expenses.

"They said this procedure is not covered under the scheme, so we had to pay."

Delays in pre-authorization and claim approval were also commonly reported particularly in emergency situations where immediate treatment was required.

"We had to wait for approval before starting treatment which caused delays."

Participants also highlighted the need for better communication and guidance from healthcare providers.

"No one clearly explained how to use insurance. We had to figure it out ourselves."

Integration with quantitative findings

The qualitative findings complement the quantitative results by providing a deeper understanding of the reasons behind the low utilization of health insurance (28%) observed in the study. While quantitative analysis identified significant associations with education, socioeconomic status, and awareness, qualitative insights revealed the underlying barriers such as lack of knowledge, administrative complexity, financial constraints, and sociocultural influences.

These findings emphasize that improving health insurance utilization in rural areas requires addressing both individual-level factors (awareness, literacy, beliefs) and system-level barriers (accessibility, administrative efficiency, service coverage).

## Discussion

This current mixed-methods study carried out in a rural field practice area in Tiruvallur district found that only 28% of the subjects used the available health insurance services, suggesting a considerable discrepancy between the availability and effective use of the insurance services. While there are instances where such utilization discrepancies are recorded in past studies in India, the significant level of underutilization witnessed in this rural community underscores the presence of context-specific challenges inherent in rural environments, including administrative unfamiliarity, informal communication channels, and restricted access to healthcare facilities [1,3,15].

The positive correlation established between education, socioeconomic factors, and insurance use implies the role played by social inequities in impacting healthcare access. Despite several past studies highlighting comparable trends, the results from the current study suggest that among rural communities, socioeconomic inequity impacts not only the ability of individuals to afford insurance but also their access to accurate sources of information [6,17]. Compared to urban areas where organized means of communication, such as media contact and institutional contact, are more available, rural communities usually depend on personal contacts, such as those with neighbors and family members [8,14].

Awareness came out strongly as one of the key enablers that influenced usage behavior. From the findings in the qualitative analysis, it became apparent that the level of awareness among the respondents was shallow and only amounted to mere awareness of the name of the schemes rather than the procedural process. This gap between shallow awareness and functional knowledge is especially critical in the context of rural areas, where illiteracy levels could hamper the conversion of awareness into usage [9,12]. It would therefore appear necessary to enhance health insurance literacy to improve usage in rural areas [8,12].

The obstacles related to administrative and procedural factors were highlighted as important discouragements for utilization. In terms of administrative procedures, the participants cited issues of documentation and a lack of clear directions in hospitals. Although the effect of bureaucracy on health-seeking behavior has been documented in past research, the current results suggest that such barriers could be even more severe among rural communities because of lower institutional awareness and fewer social networks [11,14,15]. Lack of proper guidance systems in hospitals could dissuade individuals from using insurance services, despite being covered, due to the difficulties involved.

Apart from these factors, it was also noted that financial considerations, including indirect costs like travel, medicine, and earning losses while in the hospital, have some role to play in utilization decisions. While health insurance intends to minimize direct medical expenses, the fact that indirect financial issues still exist despite having health insurance programs is indicative of inherent weaknesses in the present system [2,18]. In this respect, it becomes even more relevant in rural areas wherein people survive by earning money every day. As mentioned above, the findings of past literature have been similar to those obtained here [16,18].

Determinants like sociocultural factors gained prominence as factors impacting the health-seeking behaviors of the study population. Traditional gender roles, household decision-making processes, and cultural beliefs played a vital role in the decision-making process of using insurance services. Women and the older generation made use of male members and younger people for decision-making concerning healthcare which is a reflection of the deep-rooted sociocultural aspects influencing individual freedom and healthcare service usage [10,11]. In addition to this, stigmatization and the quality of healthcare services provided by insurance affected the behavioral decisions of the study participants [19,20].

The factors of geographical accessibility and health system-related elements also had a significant influence on healthcare utilization trends. Patients who were geographically distant from health facilities reported having low utilization trends, underscoring the importance of geographical accessibility as one of the structural determinants that affect healthcare practices [6,9]. In rural settings characterized by poor transport infrastructure and an uneven distribution of health facilities, geographical barriers could prevent people from seeking out healthcare facilities or using their insurance benefits. The results indicate the importance of investing in rural healthcare facilities and services [6,11].

The results of this study may be interpreted through theoretical models of healthcare utilization that incorporate the interplay between predisposing, enabling, and need determinants of healthcare behavior. Among predisposing determinants for the target rural population, education and socioeconomic status were found to be associated with knowledge and awareness of the insurance scheme. Meanwhile, accessibility, awareness level, and geographic proximity were found among the enabling determinants that affected utilization behavior. Finally, the need determinants included the degree of illness severity. This example shows how complicated the process of healthcare decision-making is for the residents of rural areas and why behavioral models may be relevant to interpret utilization in specific contexts [10].

The combination of quantitative and qualitative results constitutes a major strength of the current study. The quantitative results helped identify objective factors that influence utilization, whereas the qualitative results provided insight into the reasons behind the behavior and context that influenced these relationships. The discovery of administrative difficulties and sociocultural obstacles can help explain why moderate awareness does not result in corresponding levels of utilization. This combination enriches the interpretation process of the research and shows the usefulness of employing both methods to deal with difficult problems in public health related to healthcare access and utilization [1,14].

Significantly, the study has contributed to the existing body of literature by offering context-based evidence drawn from the practice of a field in the rural setting which is often an underexplored area in the study of health insurance. Most studies conducted before have been on the urban or larger administrative level which might lack a certain understanding of local cultural barriers to health insurance. The findings of the current study have shed light on specific factors related to rural settings, such as the use of informal communication channels, family decision-making, and indirect costs associated with health insurance [6,15].

The results highlight that poor access to healthcare facilities among rural communities cannot be linked to a single determinant; instead, it reflects the combined impact of a number of socioeconomic and cultural influences. In order to overcome the multifaceted barriers, there must be a holistic approach that focuses on creating community-based awareness campaigns, streamlining administrative processes, promoting healthcare facilities, and building confidence in public healthcare institutions. Contextually driven solutions will play an important role in overcoming the gap between insurance coverage and use to achieve universal healthcare in rural India [2,13].

This study identifies several limitations that may affect the interpretation of its findings. The cross-sectional design restricts the establishment of causal relationships between social determinants and health insurance utilization. Conducted in a single rural area, the results may lack generalizability. Self-reported data on insurance utilization, awareness, and expenditure could introduce recall or reporting bias. The analysis primarily employed descriptive statistics and chi-squared tests, possibly overlooking confounding factors. Future research using multivariable regression analysis could better identify independent predictors. Additionally, while purposive sampling and thematic analysis were implemented, the qualitative findings may not represent all population groups and are context-specific, limiting their transferability to other settings.

## Conclusions

From the implications of the current research study, it is clear that effective use of health insurance by rural residents involves considering some of the obstacles that stand in the way of service access for patients. The identified discrepancy in health insurance service use among the study sample is due to a number of reasons, namely, lack of awareness about health insurance, lack of understanding about eligibility criteria and benefit package details, costs associated indirectly such as travel time and wages lost, and problems associated with the actual procedures involved in obtaining care within medical facilities. It is therefore important to enhance communication at the community level, facilitate information and documentation requirements, and make the services provided by enrolled healthcare facilities accessible to rural residents. It is also essential to take into account the sociocultural factors, including family influence and preference for public health programs, among others.

## Appendices

Appendix A

Section A: Socio-demographic Details

1. Age (in completed years): _______

Classified as: ☐ 20-39 ☐ 40-60 ☐ >60

2. Gender: ☐ Male ☐ Female

3. Education level: ☐ Illiterate ☐ Primary school ☐ Secondary school ☐ Higher education

4. Occupation: ☐ Unemployed ☐ Unskilled worker ☐ Semi-skilled worker ☐ Skilled worker ☐ Professional

5. Monthly family income (₹): ₹ __________

(Used to classify socioeconomic status based on the Modified BG Prasad Scale)

6. Socioeconomic status (Modified BG Prasad Scale): ☐ Lower ☐ Middle ☐ Upper

7. Total no. of family members: ________

Section B: Health Insurance Status

8. Are you enrolled in any health insurance scheme? ☐ Yes ☐ No

9. If Yes, specify type of insurance: ☐ Government scheme ☐ Private insurance ☐ Employer-based insurance

10. Have you utilized health insurance during your last hospitalization? ☐ Yes ☐ No

Section C: Awareness of Health Insurance

11. Are you aware of health insurance schemes available in your area? ☐ Yes ☐ No

12. Source of information about insurance: ☐ Family/friends ☐ Health workers (accredited social health activist (ASHA)/auxiliary nurse midwife (ANM)) ☐ Media (TV/radio) ☐ Others (specify): __________

Section D: Health-Related Information

13. Type of illness during the last hospitalization: ☐ Major illness ☐ Minor illness

14. Distance to the nearest healthcare facility: ☐ <5 km ☐ ≥5 km

Section E: Reasons for Non-utilization

(If insurance is not utilized)

15. Main reason for not utilizing insurance: ☐ Lack of awareness ☐ Procedural difficulties ☐ Lack of documents ☐ Preference for direct payment ☐ Other (specify): __________

Section F: Out-of-Pocket Expenditure

16. Approximate total out-of-pocket expenditure during hospitalization: ₹ __________

Appendix B

Section A: Awareness

1. Can you tell me what you know about health insurance schemes available in your area?

2. How did you first hear about health insurance?

3. What do you think are the benefits of health insurance?

Section B: Experience With Insurance

4. Can you describe your experience during your last hospitalization?

5. Were you able to use health insurance during hospitalization? Why or why not?

6. What challenges did you face while trying to use insurance?

Section C: Administrative Barriers

7. Did you face any difficulties with documentation?

8. Was the process of using insurance easy or difficult to understand?

9. Did hospital staff explain the insurance process clearly?

Section D: Financial Issues

10. Did you have to spend money despite having insurance?

11. What expenses were not covered by insurance?

12. Did loss of daily wages affect your decision to stay in the hospital?

Section E: Sociocultural Influences

13. Who usually makes healthcare decisions in your household?

14. Do family members influence the decision to use insurance?

15. Are there any social beliefs or stigma related to using government schemes?

Section F: Health System Barriers

16. Were there delays in receiving treatment due to insurance procedures?

17. Were all treatments covered under insurance?

18. Were the empanelled hospitals easily accessible?

Section G: Suggestions

19. What changes would help people in your village use health insurance more easily?
